# Training-induced increase in Achilles tendon stiffness affects tendon strain pattern during running

**DOI:** 10.7717/peerj.6764

**Published:** 2019-04-24

**Authors:** Amelie Werkhausen, Neil J. Cronin, Kirsten Albracht, Gøran Paulsen, Askild V. Larsen, Jens Bojsen-Møller, Olivier R. Seynnes

**Affiliations:** 1 Department of Physical Performance, Norwegian School of Sport Sciences, Oslo, Norway; 2 Neuromuscular Research Centre, Faculty of Sport and Health Sciences, University of Jyväskylä, Jyväskylä, Finland; 3 Institute of Biomechanics and Orthopedics, German Sport University Cologne, Cologne, Germany; 4 Department of Medical Engineering and Technomathematics, Aachen University of Applied Sciences, Aachen, Germany; 5 The Norwegian Olympic and Paralympic Committee and Confederation of Sports, Oslo, Norway

**Keywords:** Achilles tendon, Stiffness, Running, Tendon properties, Architectural gear ratio, Gastrocnemius, Soleus

## Abstract

**Background:**

During the stance phase of running, the elasticity of the Achilles tendon enables the utilisation of elastic energy and allows beneficial contractile conditions for the triceps surae muscles. However, the effect of changes in tendon mechanical properties induced by chronic loading is still poorly understood. We tested the hypothesis that a training-induced increase in Achilles tendon stiffness would result in reduced tendon strain during the stance phase of running, which would reduce fascicle strains in the triceps surae muscles, particularly in the mono-articular soleus.

**Methods:**

Eleven subjects were assigned to a training group performing isometric single-leg plantarflexion contractions three times per week for ten weeks, and another ten subjects formed a control group. Before and after the training period, Achilles tendon stiffness was estimated, and muscle-tendon mechanics were assessed during running at preferred speed using ultrasonography, kinematics and kinetics.

**Results:**

Achilles tendon stiffness increased by 18% (*P* < 0.01) in the training group, but the associated reduction in strain seen during isometric contractions was not statistically significant. Tendon elongation during the stance phase of running was similar after training, but tendon recoil was reduced by 30% (*P* < 0.01), while estimated tendon force remained unchanged. Neither gastrocnemius medialis nor soleus fascicle shortening during stance was affected by training.

**Discussion:**

These results show that a training-induced increase in Achilles tendon stiffness altered tendon behaviour during running. Despite training-induced changes in tendon mechanical properties and recoil behaviour, the data suggest that fascicle shortening patterns were preserved for the running speed that we examined. The asymmetrical changes in tendon strain patterns supports the notion that simple in-series models do not fully explain the mechanical output of the muscle-tendon unit during a complex task like running.

## Introduction

The integrated function of tendon and muscle plays an important role for force production during locomotion. The viscoelastic nature of the Achilles tendon enables energy conservation by uncoupling muscle fascicle behaviour from that of the muscle–tendon unit (MTU), which allows beneficial contractile conditions for the triceps surae muscles and that favours economic force production (Roberts et al., 1997). During running, gastrocnemius fascicles shorten throughout the stance phase, while the Achilles tendon and other series elastic elements initially stretch and recoil during the following push-off phase (Lichtwark, Bougoulias & Wilson, 2007). It follows that changes in Achilles tendon stiffness potentially have important consequences for both tendon elastic energy storage and muscle contractile behaviour during running.

While research has focused on how muscle adaptations influence force production, a better understanding of the role of tendon stiffness during running is required, especially when considering the significant changes in tendon mechanical properties that occur with loading (Wiesinger et al., 2015), ageing (McCrum et al., 2018) or injury (Obst et al., 2018). Cross-sectional comparison studies have shown differences in Achilles tendon stiffness between athletes with contrasting running specialisation (Arampatzis et al., 2007b) or performance (Kubo et al., 2015), but longitudinal data supporting a causative link are scarce (Albracht & Arampatzis, 2013). Simulation studies suggest that varying tendon stiffness affects muscle contractile behaviour, muscle mechanical work and metabolic energy consumption (Lichtwark & Wilson, 2007; Lichtwark & Wilson, 2008). On the basis of a two-dimensional model of the Achilles tendon in series with the gastrocnemius muscle, a change in tendon stiffness would affect the functional range of fascicles (i.e., fascicle operating length and shortening velocity), which would in turn alter the conditions of muscle work production. Contrary to this assumption, Albracht & Arampatzis (2013) did not find any alteration in gastrocnemius fascicle behaviour during running after a training-induced increase in Achilles tendon stiffness. However, in this study, the calculated strain of the series elastic elements (SEE) during running was unaltered after training, despite the reduction in tendon strain observed during isometric contractions. This discrepancy may have been caused by the complexity and number of tissues reflected by SEE length calculations. SEE length may not reflect tendon strain accurately enough because it involves all other elastic structures within the MTU, which may not be arranged completely in series with the muscle and may adapt differently from the tendon. Specific measurements of Achilles tendon length may be necessary to ascertain that this type of intervention does not affect tendon strain patterns during running. The lack of adjustment in gastrocnemius fascicle behaviour found by these authors is nonetheless noteworthy, and may be attributable to the bi-articular nature of this muscle. While lengthening of the gastrocnemius MTU is partly offset by knee flexion during the first part of the stance phase (i.e., 0–40%), the relatively greater lengthening of the soleus MTU and SEE (Lai et al., 2018) caused by ankle dorsiflexion suggests that length changes in this muscle may be more affected by a training-induced increase in tendon stiffness.

We investigated how a training-induced increase in Achilles tendon stiffness affects the strain patterns of the Achilles tendon and of the fascicles of gastrocnemius medialis and soleus muscles during running. We hypothesized that, while joint kinematics and kinetics would remain similar before and after training (Albracht & Arampatzis, 2013), a training-induced increase in Achilles tendon stiffness would reduce Achilles tendon strain and recoil during running. Additionally, we expected that the change in tendon properties would decrease muscle fascicle shortening and shortening velocity, primarily in the soleus, owing to the mono-articularity of this muscle.

## Materials & Methods

### Subjects and experimental design

The experiment was performed on 21 recreationally-active adults, as a part of a larger study partly published elsewhere (Werkhausen et al., 2018). Volunteers were excluded from the study if they reported any injury or a history of systematic plantar flexor strength training. Eleven subjects were assigned to the training group (height 174 cm, body mass 70 kg, age 26 years, five men and six women) and ten subjects served as controls (height 178 cm, body mass 73 kg, age 30 years, six men and four women). All subjects were fully informed about the experiment and provided written informed consent to participate in this study. The protocol was approved by the Ethical Committee of the Norwegian School of Sport Sciences (14-220817) and conducted in accordance with the Declaration of Helsinki.

The training group took part in a resistance training program focused on plantar flexor muscles of the right leg for ten weeks, while the control group did not change their daily activities during this period. All subjects were tested similarly before and after the training period. All tests were performed barefoot and on the right leg only. The warm-up for the testing sessions consisted of 5 min barefoot running on a treadmill at individual preferred speed. The testing procedure included two experiments using ultrasound, kinematic and kinetic measurements synchronized by a trigger signal sent by the ultrasound device. Firstly, Achilles tendon stiffness, plantar flexion strength and resting gastrocnemius muscle architecture were measured. Secondly, *in vivo* muscle–tendon behaviour during running at preferred speed (2.8 ± 0.4 ms^−1^ and 2.3 ± 0.2 ms^−1^ for the training group and the controls, respectively, as determined during the first test) were examined. The running test was performed twice, to acquire images of the muscle–tendon junction and of the muscle fascicles separately.

### Training program

The training group performed four sets of ten repetitions, unilateral, isometric plantar flexion contractions three times per week for ten weeks. Both legs were trained alternately, whereas training of the left leg was optional when subjects experienced back pain due to the unilateral loading. The exercise was always preceded by a five-minute easy warm-up on a cycle ergometer. As the aim was to increase Achilles tendon stiffness while minimising adaptations of muscle architecture and strength, fixed-end contractions were performed explosively and maintained for one second followed by five seconds of rest (Balshaw et al., 2016; Massey et al., 2018). Plantar flexion training was performed in a standing position in a custom-built rig (Werkhausen et al., 2018). The training device was adjusted for every subject so that the ankle was in anatomical position (i.e., foot perpendicular to the tibia) during the contractions. The maximum plantar flexion force for the training task was measured during the first training session of every week to determine the target force of 80% of the maximum force used in the training. Visual feedback of the instantaneous force was provided to the subjects during training.

### Muscle-tendon unit properties

Lying prone with the knee fully extended and the hip and ankle joint in the anatomical position, the gastrocnemius medialis muscle was imaged at the mid-belly using ultrasound with a transducer length of 60 mm (LS 128; Telemed, Vilnius, Lithuania). Fascicle length, pennation angle and muscle thickness were analysed offline (ImageJ, National Institutes of Health, Bethesda, USA). Fascicle length was segmented manually, as the distance between the upper and lower aponeuroses along a direction parallel to the visible portions of fascicles. Pennation angle was defined as the orientation of fascicles relative to the deep aponeurosis.

In the same position, subjects were then securely strapped into a dynamometer and asked to perform isometric plantar flexions (IsoMed 2000; D&R Ferstl GmbH, Hemau, Germany). The highest torque of two attempts was set as the maximum torque. Subsequently, subjects were asked to perform ramp contractions, while ultrasound scans (LS 128; Telemed, Vilnius, Lithuania, 96 elements, 80 Hz) of the gastrocnemius myotendinous junction, plantar flexion torque (sampled at 600 Hz) and marker trajectories (Qualisys, Gothenburg, Sweden, sampled at 120 Hz) were recorded simultaneously to estimate tendon stiffness. Using visual feedback, subjects were instructed to increase torque at a loading rate of 100 N m s^−1^ up to 90% of the individual maximum torque. An ultrasound gel pad was placed between the transducer and the skin to ensure consistent transmission of sound waves when the muscle was bulging. A bidirectional second order low-pass Butterworth filter with a cut-off frequency of 15 Hz was used to smooth torque, marker trajectory and ultrasound data.

A four-camera motion capture system was used to record the position of a triad of reflective markers, which were rigidly attached to the ultrasound probe. Using prior calibration, the position tracked in the two-dimensional ultrasound image was projected into the three-dimensional coordinate system of the laboratory. The position of the Achilles tendon insertion onto the calcaneus was determined with ultrasonography and identified externally with a reflective marker. Ultrasound images were analysed offline via semi-automatic tracking of a fascicle insertion as close as possible to the gastrocnemius myotendinous junction (Tracker 4.95, http://physlets.org/tracker/) to define the proximal end of the tendon. Achilles tendon length was subsequently calculated as the straight distance between the myotendinous junction and the Achilles tendon insertion in the three-dimensional coordinate system of the laboratory.

The measured joint torque was corrected for unavoidable ankle joint rotation by recording the position of reflective markers on the medial malleolus and the footplate of the dynamometer (Arampatzis et al., 2005). The torque contribution of the triceps surae (91%) was estimated from normative data including the relative volume, optimal fibre length and moment arm of this muscle group, which was reported to be generally consistent across joint angles and subjects (Dick, Arnold & Wakeling, 2016, Fig. S3). Achilles tendon moment arm was estimated as the mean perpendicular distance between the line of action of the Achilles tendon and the mid-point of the mediolateral distance between the malleoli. Subsequently, Achilles tendon force was calculated by dividing the corrected torque by the Achilles tendon moment arm.

Tendon stiffness was obtained for every subject and test session from the mean force–elongation relationship of three trials. Five trials were recorded for this purpose, and the trials yielding the lowest and highest stiffness values were excluded. The mean force–elongation curves were first fitted with a third-order polynomial and stiffness was calculated as the slope of the linear portion between 50% and 80% of the maximum individual force. All strain measurements were obtained for each individual at 80% of the force produced during the pre-training test.

### Muscle-tendon mechanics during running

Data were collected while the subjects were running at their preferred speed on one force plate of an instrumented treadmill (Force-Link, Motek, Netherlands, sampling at 1,500 Hz). Force data were synchronized with three dimensional kinematic data recorded using at least 12 cameras of a motion capture system (Qualisys, Gothenburg, Sweden, sampled at 300 Hz). Raw data were filtered using a bidirectional second-order low-pass Butterworth filter with a cut-off frequency of 15 Hz. Reflective markers were placed according to a modified Cleveland Clinics marker set to define pelvis, thigh, shank and foot segments and to track their movement during motion trials (left and right anterior and posterior iliac spine; right medial and lateral epicondyles; right medial and lateral malleoli; posterior calcaneus and first, second and fifth metatarsals; clusters of four markers to track the right thigh and shank segments). Ankle and knee joint angles and moments were calculated using inverse kinematics and kinetics analysis performed in Visual3D (C-Motion, Germantown, MD, USA). The MTU length of gastrocnemius medialis and soleus was estimated using joint angle data and shank length defined as the distance from the lateral malleolus to the lateral epicondyle (Hawkins & Hull, 1990).

During running, the ultrasound transducer was secured over the myotendinous junction or the muscle belly of the medial gastrocnemius as appropriate using self-adhesive bandages. Ultrasound images were sampled at 80 Hz (60 mm, 96 elements, LS 128 Telemed, Vilnius, Lithuania). Achilles tendon length and Achilles tendon forces were estimated according to the same method as described for prone measurements. Software for the semi-automated tracking of fascicles in ultrasound images was used to analyse fascicle length and pennation angle during the running trials (Cronin et al., 2011; Farris & Lichtwark, 2016). Similar to resting architecture measurements, pennation angle was calculated between the fascicle and the line of action of the muscle. Subsequently, muscle thickness was estimated by multiplying fascicle length by the sine of pennation angle.

### Data analysis and statistics

Fascicle and MTU length data were differentiated with respect to time to calculate their respective velocities. The influence of fascicle rotation on fascicle and muscle shortening was quantified using a modified version of the architectural gear ratio described in Brainerd & Azizi (2005). Architectural gear ratio was calculated for gastrocnemius and soleus, similarly to Hollville et al. (2018), as the ratio between the projected muscle length change along the axis of the deeper aponeurosis (calculated as fascicle length multiplied by the cosine of the pennation angle (as in Fukunaga et al., 2001)) and fascicle length change during the stance phase. Hereafter this is referred to as architectural gear ratio stance (AGRs). Due to variability in duration of the ground contact, all data were resampled to an equal number of 101 data points during the stance phase. For each subject, ten steps were initially included in the analysis. Individual steps were excluded when data differed more than two times standard deviation from the rest of the steps as determined by visual inspection so that data were averaged over eight steps for the final analysis for each subject. Due to insufficient image quality, ultrasound data on soleus fascicles for three subjects of the training group were discarded (*n* = 8) and data on gastrocnemius myotendinous junction were discarded for two subjects of the training group ( *n* = 9) and three controls (*n* = 7).

Length changes, peak length and peak velocity values of the relevant variables were compared with a two-way ANOVA design using the factors time (pre vs. post training) and group (training vs. control group). Bonferroni post-hoc tests were used where significant interaction or main effects were detected (Prism, GraphPad Software Inc., La Jolla, CA, USA). All variables were analysed during the stance phase. Statistical significance was set to *P* > 0.05.

## Results

### Muscle tendon properties and strength

The results for muscle–tendon properties and strength have been reported in detail in a previous study (Werkhausen et al., 2018) and are presented in Table 1. While isometric 1RM torque, measured during the training exercise, increased by 39% (*P* < 0.01) between the first and the last training week, the maximal torque measured in the dynamometer increased by 15% (*P* < 0.01) in the training group. Tendon stiffness increased by 18% (*P* < 0.01), although tendon strain did not decrease significantly (*P* = 0.18). No changes were found in the control group for maximal plantar flexion torque, Achilles tendon stiffness or strain, and muscle architecture measurements (*P* > 0.05 for all comparisons).

**Table 1:** Plantarflexion torque, muscle architecture and Achilles tendon (AT) stiffness in the training group and the control group measured before (pre) and after (post) the training intervention.

	Training group		Control group	
	Pre	Post	Pre	Post
Torque [N m]^b^	172 ± 50	198 ± 51^c^	170 ± 51	180 ± 62
Lf GM [mm]	89.2 ± 13.3	90.7 ± 16.1	84.2 ± 16.1	84.0 ± 10.4
PA GM [°]	18.1 ± 1.8	19.0 ± 2.0^c^	18.4 ± 1.1	18.3 ± 1.7
Thickness GM [mm]^b^	23.7 ± 3.5	25.0 ± 3.8^c^	23.1 ± 3.8	23.5 ± 3.6
AT stiffness [N mm-1]^a^^,^^b^	397 ± 146	459 ± 147^c^	399 ± 193	400 ± 212
AT strain [mm]	4.4 ± 1.1	4.1 ± 0.5	4.0 ± 1.5	3.9 ± 1.5

**Notes.**

Values are means ± sd.

GM gastrocnemius medialis SOL soleus Lf length of fascicle PA pennation angle

a *P* < 0.05 interaction effect.

b *P* < 0.05 main effect of time.

c *P* < 0.05 comparing pre- and post-intervention test (Werkhausen et al., 2018).

### Effect of training on running kinematics and kinetics

**Figure 1:**
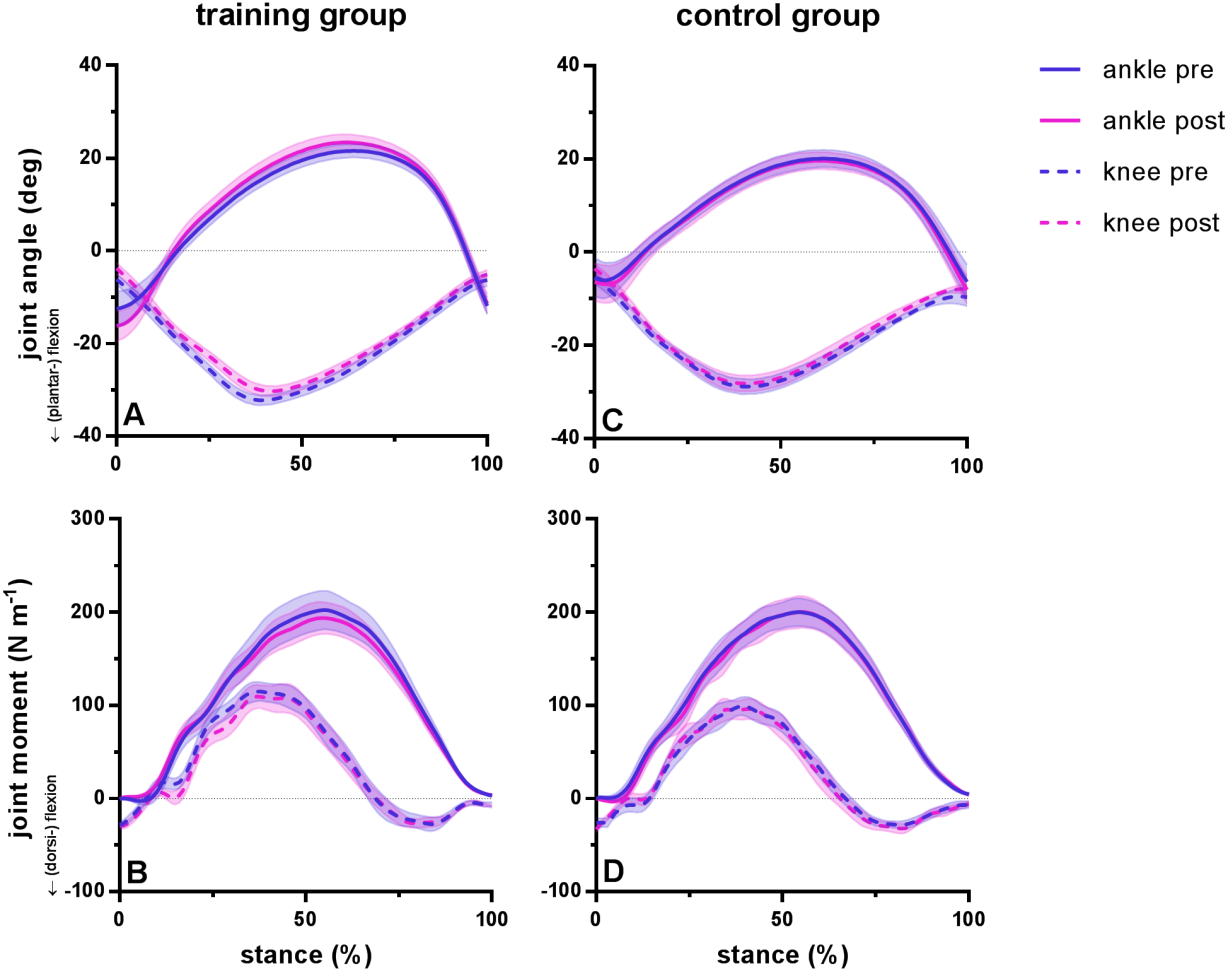
Group mean ankle and knee joint angles and moments during the stance phase of barefoot running at preferred speed for the training group (A–B) and the control group (C–D). Time series are normalized to 101 points. Values are means ± s.e.m.

Running kinematics and kinetics were statistically similar pre and post training for subjects in both groups. Duty factor did not vary before and after training in either group but was shorter in the training group compared to the control group. During the stance phase, maximum dorsiflexion range (pre 34 ±12°, post 40 ± 10°; *P* = 0.08) and subsequent plantar flexion range (pre 34 ± 4° , post 35 ± 7°; *P* = 0.21) did not differ before and after training in the training group. Similarly, knee range of motion remained similar after training for maximum flexion (pre 26 ± 2°, post 27 ±2°; *P* = 0.16) and maximum extension (pre 26 ±5°, post 25 ± 4°; *P* = 0.99) during the stance phase. No significant differences were found for peak moments of the ankle joint (pre 206 ± 70 Nm, post 197 ± 57 Nm; *P* = 0.77) or knee joint (pre 126 ± 36 Nm, post 122 ± 48°; *P* = 0.93). Ankle range of motion was different between groups, whereas joint kinematics remained similar between tests for the control group, with a coefficient of variation between 4 and 5% for ankle and knee flexion and 7% for ankle dorsiflexion and knee extension. Ankle and knee joint angles and moments during the stance phase during running are presented in Fig. 1 for the training group (A–B) and the control group (C–D) before and after training.

**Figure 2:**
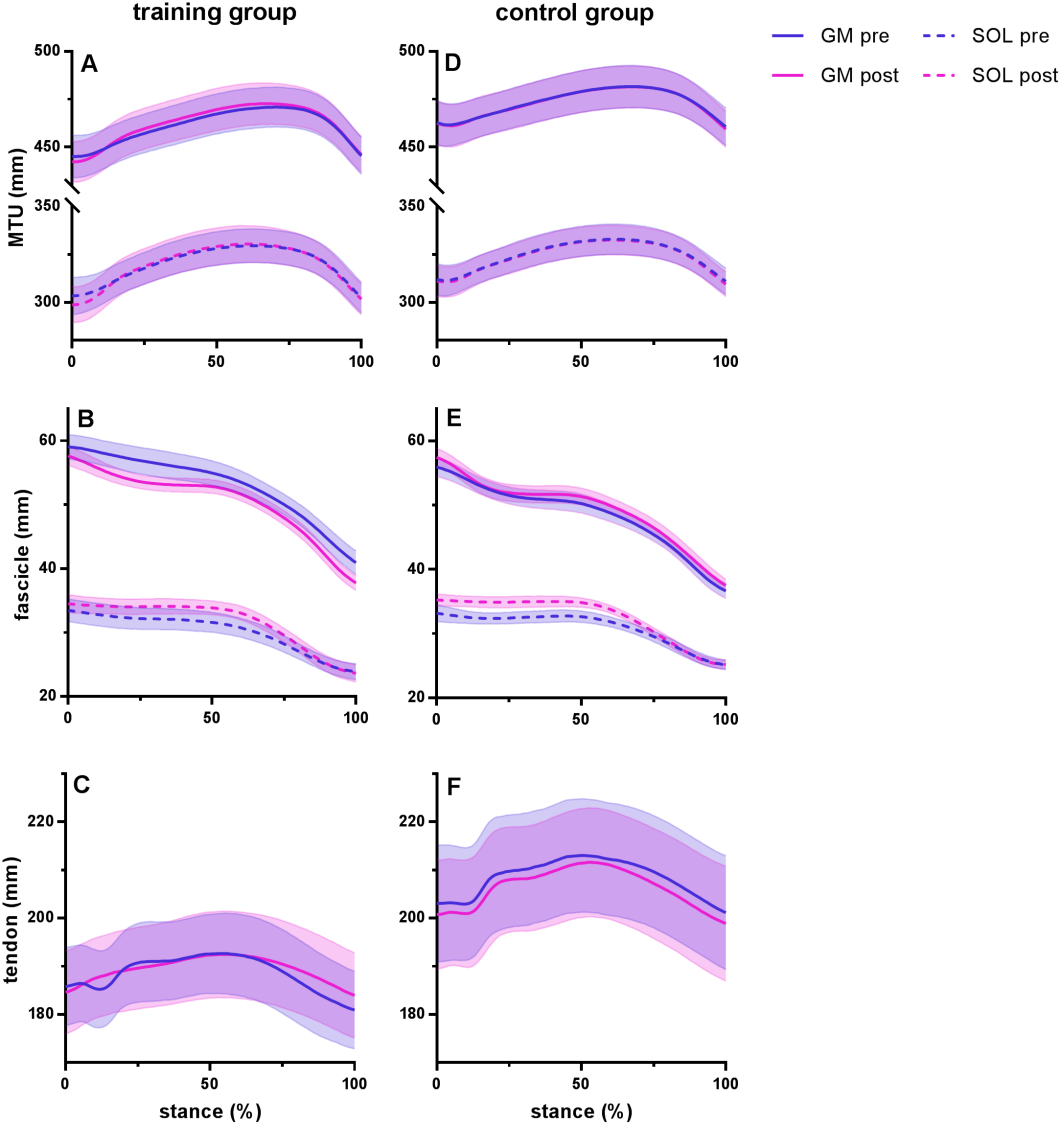
Muscle-tendon unit (MTU), fascicle and tendon length in the training group (A–C) and the control group (D–F) for gastrocnemius medialis (GM) and soleus (SOL) before (pre) and after (post) training. Tendon length was measured between gastrocnemius medialis muscle-tendon junction and Achilles tendon insertion. Time series are normalized to 101 points. Values are means ± s.e.m.

### Effect of training on muscle–tendon interaction during running

**Figure 3:**
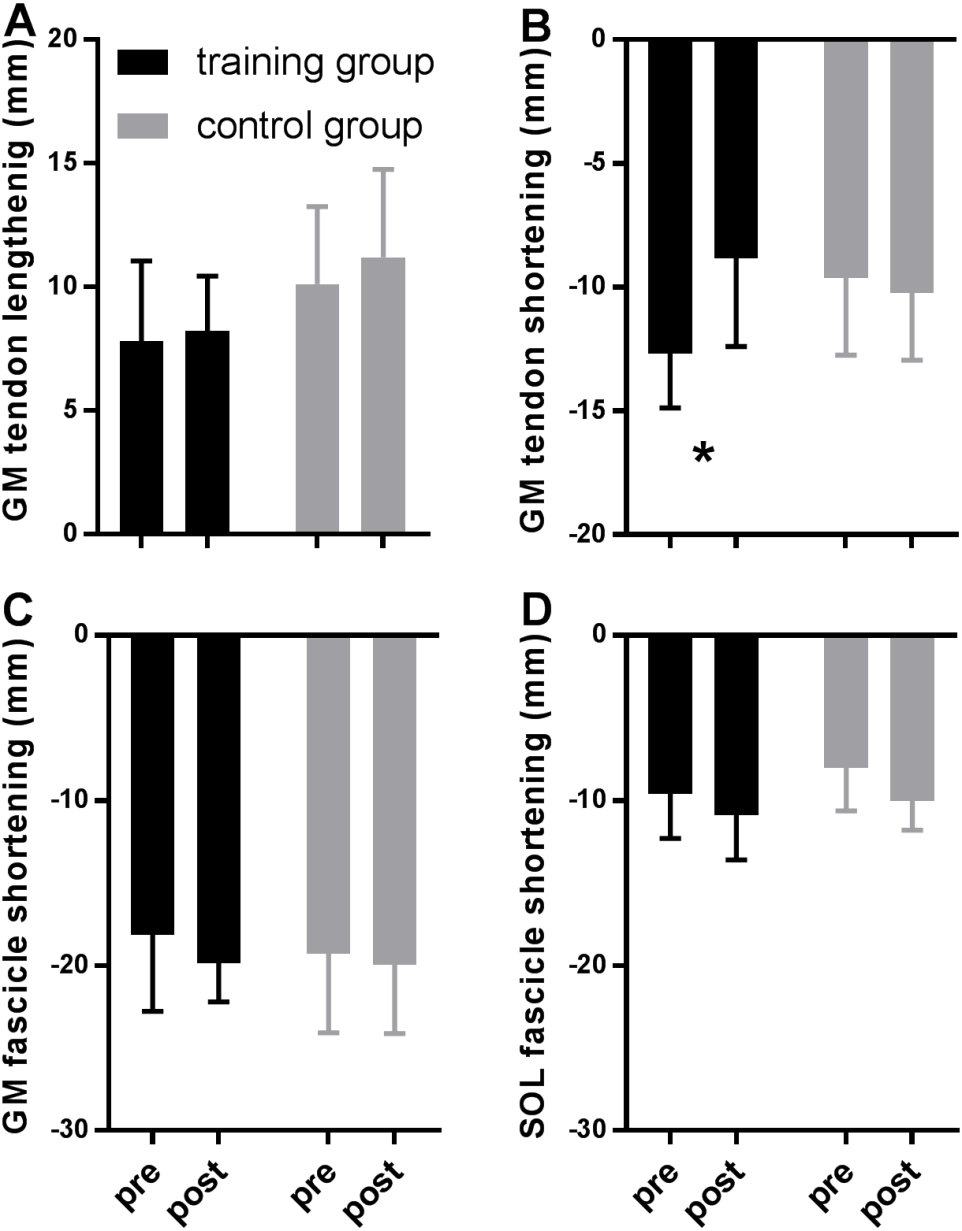
Tendon (A–B) and fascicle (C–D) measures during the stance phase of running compared between tests pre- and post-training in the training (black) and the control group (grey). Data are means ± s.d., * *P* < 0.05 when comparing pre vs. post intervention.

Figure 2 shows MTU, muscle fascicle and Achilles tendon length during the stance phase of running pre and post training for the training group (A–C) and the control group (D–F). The typical MTU stretch-shortening pattern during running did not differ in amplitude after training (Figs. 2A & 2D) in the gastrocnemius (Interaction *P* = 0.11 and *P* = 0.25 for stretch and shortening, respectively) or the soleus (Interaction *P* = 0.11 and *P* = 0.76 for stretch and shortening, respectively). Muscle fascicles shortened throughout the stance phase, but neither shortening amplitude nor peak fascicle velocity differed after training in either muscle (Table 2, Figs. 2B & 2E, 3C & 3D). However, the rotation of fascicles about their deeper insertion during the stance phase, i.e., the change in pennation angle, tended to increase in gastrocnemius (Interaction *P* = 0.10, Time *P* = 0.04, Post-hoc test comparing pre vs. post intervention, *P* = 0.01 for the training group and *P* >0.99 for the control group), but not soleus (non-significant interaction or time effect). Accordingly, there was no significant interaction (*P* = 0.11), but a main effect of time for architectural gear ratio of gastrocnemius (*P* = 0.05), and post-hoc tests indicated a trend for a significant increase of this parameter in the trained group (*P* = 0.01). There was no interaction or main effect for soleus gear ratio (Table 2). Similarly to the MTU, no difference was observed after training for Achilles tendon stretch (Interaction *P* = 0.69). However, we found an interaction effect for Achilles tendon shortening (*P* = 0.02). Post-hoc comparisons showed that tendon recoil was reduced by 30% in the training group (*P* < 0.01), whereas this variable did not change in the controls (Table 2, Figs. 2C, 3B). Despite shorter MTU lengths in the training group compared to the controls, there were no differences between groups in tendon and fascicle behaviour.

**Table 2:** Relevant variables for muscle-tendon unit (MTU), Achilles tendon (AT) and fascicles (F) of gastrocnemius (GM) and soleus (SOL) behavior during the stance phase of running at preferred speed.

		**Training group**			**Control group**		
		**pre**	**post**	**diff**	**pre**	**post**	**diff**
**Lengthening (mm)**	**MTU GM**	26 ± 10	30 ± 9	5 ± 7	19 ± 7	19 ± 7	0 ± 2
	**MTU SOL**	26 ± 10	32 ± 9	6 ± 8	21 ± 7	22 ± 7	0 ± 2
	**AT**	8 ± 3	8 ± 2	0 ± 4	10 ± 3	11 ± 4	1 ± 2
**Shortening (mm)**	**MTU GM**	26 ± 4	27 ± 7	1 ± 4	21 ± 8	22 ± 7	1 ± 3
	**MTU SOL**	27 ± 4	29 ± 8	2 ± 5	22 ± 9	23 ± 7	1 ± 3
	**AT**^a^	13 ± 2	9 ± 4^c^	4 ± 4	10 ± 3	10 ± 3	1 ± 2
	**F GM**	18 ± 5	20 ± 2	2 ± 5	19 ± 5	20 ± 4	1 ± 4
	**F SOL**	10 ± 3	11 ± 3	1 ± 2	8 ± 3	10 ± 2	2 ± 3
**Shortening vel (mm s**^−1^)	**F GM**	179 ± 51	205 ± 41	26 ± 50	163 ± 48	166 ± 17	3 ± 41
	**F SOL**	108 ± 41	133 ± 41	25 ± 40	95 ± 22	114 ± 17	19 ± 25
**AGRs**	**GM**^b^	1.06 ± 0.03	1.09 ± 0.03^c^	0.03 ± 0.04	1.07 ± 0.03	1.08 ± 0.02	0.0 ± 0.02
	**SOL**	1.06 ± 0.11	1.05 ± 0.11	0.01 ± 0.08	1.12 ± 0.09	1.15 ± 0.09	0.03 ± 0.07
**Change in pennation(°)**	**GM**^a^^,^^b^	7.7 ± 3.4	10.2 ± 2.2^c^	2.5 ± 2.7	9.4 ± 3.2	9.7 ± 2.0	0.3 ± 2.5
	**SOL**	8.8 ± 2.8	9.8 ± 4.1	1.0 ± 3.1	9.6 ± 3.1	12.6 ± 3.8	2.9 ± 4.4
**Thickness change (mm)**	**GM**	0.8 ± 0.9	0.6 ± 1.0	0.1 ± 1.1	0.7 ± 0.7	0.6 ± 0.6	0.1 ± 0.7
	**SOL**	3.2 ± 2.9	3.7 ± 2.2	0.5 ± 1.4	2.5 ± 1.0	3.1 ± 1.4	0.5 ± 1.4
**Force (N)**	**AT**	4315 ± 1646	4041 ± 1331	274 ± 1142	3632 ± 765	3765 ± 917	133 ± 279

**Notes.**

Values are means ± sd.

a *P* < 0.05 interaction effect.

b *P* < 0.05 main effect of time.

c *P* < 0.05 comparing pre- and post-intervention test.

diff absolute difference between pre- and post- intervention test

Architectural gear ratio during stance (AGRs) was calculated as the ratio between the projected fascicle length change (fascicle length multiplied by the cosine of pennation angle) and fascicle length change during the stance phase.

## Discussion

The present study examined the effect of a training-induced increase in Achilles tendon stiffness on muscle–tendon mechanics during running. After the 10-week training intervention, isometric MVC of the plantar flexor muscles increased by 15% and Achilles tendon stiffness increased by 18%. Gastrocnemius muscle thickness and pennation angle increased (5% each), but fascicle length remained unchanged (Table 2). Contrary to our predictions, tendon stretch during stance was not reduced after training, but tendon recoil was reduced. In accordance with previous findings (Lai et al., 2018), fascicles of the monoarticular soleus muscle displayed a lower shortening velocity than the gastrocnemius during the stance phase. Neither muscle seemed to present different fascicle lengthening patterns after training. None of the examined parameters changed in the control group, supporting the notion that these findings resulted from training adaptations.

### Effect of training on muscle–tendon properties

The effect of training on muscle architecture and tendon stiffness is discussed in greater detail elsewhere (Werkhausen et al., 2018). Overall, the intervention successfully increased the stiffness of the Achilles tendon while avoiding changes in resting fascicle length. The latter was of particular importance to verify the hypothesized influence of in-series elasticity on fascicle behaviour. The 18% increase in Achilles tendon stiffness was within the range reported in other studies (Wiesinger et al., 2015). Bearing in mind the short time under tension during exercise (total of 40 s per session), with the intent to limit adaptations in muscle architecture, greater increases in tendon stiffness seem likely with larger exercise stress or strain (as e.g., in Arampatzis, Karamanidis & Albracht (2007a) or Kongsgaard et al. (2007)). We can nonetheless speculate that the observed stiffening of the whole Achilles tendon would apply to the so-called free tendon and possibly to other collagenous structures, such as aponeuroses. Surprisingly, tendon strain during isometric contractions was not reduced significantly after training, which may be a consequence of the relatively low force produced during this test, or the lack of sensitivity of in vivo tendon strain and slack length measurements (Seynnes et al., 2015). Changes in the toe region of the force–elongation relationship caused by the training intervention could also have shifted the toe limit (strain at the onset of the linear region) towards longer lengths, which could explain the lack of strain reduction. The other main effect of the training intervention was the increase in plantar flexion force and associated hypertrophy, as indicated by small increases in gastrocnemius pennation angle and thickness.

### Similar tendon strain, but reduced recoil during running

Consistent with isometric tests, but contrary to our hypothesis, the tendon stretch occurring during the first part of the stance phase was not reduced after training, in spite of the greater stiffness. The same methodological limitations mentioned for the isometric tests (i.e., measurement sensitivity) may contribute to explaining this result. However, all other parameters being equal, the estimated tendon forces produced under the present running conditions would be expected to reduce tendon elongation by about 19% after training (estimation based ratios of mean tendon force to mean tendon stiffness of the training group, before and after training). With a coefficient of variation of 10% (calculated from the control group data), the sensitivity of the method used to calculate tendon length during running is deemed sufficient to detect such a reduction in tendon elongation. Yet, in agreement with our results, Albracht & Arampatzis (2013) previously found no change in SEE strain, which they attributed to increased forces and joint moments. However, in our study, GRF and ankle and knee joint moments were similar after training, suggesting that the force applied to the Achilles tendon during stance did not increase. An alternative explanation for the unchanged longitudinal stretch of the tendon could be related to the deformation pattern of the aponeuroses. Biaxial strains have been shown to occur in the proximal part of the human Achilles tendon and the aponeurosis, and to influence the longitudinal stiffness of aponeuroses in animals (Azizi & Roberts, 2009). Biaxial loading may have the same effect upon the sheet-like proximal part of the tendon from which they arise. Hence, a change in muscle contractile behaviour (discussed below) and a reduction in transverse strain of the aponeurosis and the proximal region of the Achilles tendon may have reduced the longitudinal stiffness of these structures, resulting in a similar stretch of the whole tendon during running after training. Similar to unchanged strain during isometric contractions, it may be speculated that changes in tendon slack length and the force–elongation relationship of the toe region influenced tendon strain. This hypothesis would imply that conditions to test tendon properties should more closely match the locomotor task in which stiffness is expected to play a role. For instance, the length of the gastrocnemius MTU may influence aponeurosis stiffness, as previously shown in the tibialis anterior (Raiteri, Cresswell & Lichtwark, 2018). Joint angle and contractile conditions could affect stiffness measurements more than previously thought.

In contrast with its elongation, the recoil of the Achilles tendon during the late stance phase was reduced after training. A recoil reduction seems compatible with the hypothesised effect of an increased tendon stiffness but seems counterintuitive, considering the unchanged elongation and forces produced during the stance phase. The reduction in tendon recoil alone would suggest a lower elastic energy return during push-off, but we expect the greater tendon stiffness to have in part compensated this effect. In fact, the similar joint moments measured during this phase after training are compatible with a similar elastic energy return. As discussed above, methodological shortcomings could have affected the results, since similar limitations would be expected for strain and recoil measurements. For example, estimating tendon as a straight line may underestimate tendon length changes when the joint angle changes, while this effect may be similar for stretch and recoil (Stosic & Finni, 2011).

### Similar fascicle shortening during running

Contrary to predictions based on a two-dimensional muscle–tendon model, we did not observe any post-training difference in changes in gastrocnemius or soleus fascicle length and velocity during stance with increased tendon stiffness. This result is in line with a previous study, which did not find differences in gastrocnemius fascicle shortening pattern during running after 14 weeks of isometric plantar flexion training (Albracht & Arampatzis, 2013).

Additional analyses of contractile behaviour confirmed the conclusions based on fascicle length since the increases in architectural gear ratio and pennation angle during stance seen after training (and not in the control subjects) did not reach significance either (interactions *P* = 0.11 and *P* = 0.10, respectively). We note, however, that post-hoc tests suggested a consistent tendency for these two parameters, which may have been lost to a lack of statistical power (see *Methodological considerations* section). An increased gear ratio would have been congruous with the present changes in stiffness of the tendinous tissue, since differences in AGR have typically been linked to force levels (Azizi, Brainerd & Roberts, 2008; Dick & Wakeling, 2017) or by extension, to the stiffness of the connective tissue placed in series with muscle fascicles (Eng & Roberts, 2018). In situ data have shown, with a drastic protocol consisting of aponeurosis incisions, the influence of radial stiffness on gearing at relatively high forces (Eng & Roberts, 2018). Albeit speculative, the training intervention may also have stiffened the aponeurosis and tendon radially (see discussion above), which would in turn affect AGR.

Interestingly, our data show that the soleus fascicle shortening pattern also did not change after training. Methodological difficulties prevented us from measuring the relative strains of the whole Achilles tendon and its free portion, but previous authors have shown that SEE of the soleus undergoes a greater stretch than that of the gastrocnemii during stance (Lai et al., 2018). This is consistent with measurements of tendon displacement during isometric plantar flexions at different joint angles (Clark & Franz, 2018). Yet the hypothesised greater sensitivity of soleus fascicle lengthening pattern to changes in tendon stiffness was not seen in the present study. An insufficient increase in stiffness of the free tendon to reduce strain appreciably after training could explain the unchanged soleus fascicle shortening. The strain of the shorter free tendon may be less susceptible to the induced changes in stiffness than the whole Achilles tendon. The effect of resistance training on the free tendon has to our knowledge never been reported, and further research is required to assess the effects of training on stiffness and strain of the free tendon specifically. Alternatively, changes in bi-axial loading of the soleus aponeurosis may also have occurred after training, preserving the contractile conditions of the fascicles and the important force production capacity of this muscle. However, the lack of change in soleus AGRs refutes this interpretation.

### Methodological considerations

Certain limitations inherent to the present methodology should be further discussed. The muscle hypertrophy induced by the training intervention was measured when the ankle joint was held at 90°. While this method is widely used in the literature, recent evidence indicates that inter-individual differences in tendon slack length likely influence architecture measurements (Aeles et al., 2017). We acknowledge this possibility, although its impact is deemed limited here, because of the repeated-measure design and of the negligible changes in tendon stiffness at the tension level induced in this position. To decrease the influence of noise on the ultrasound data in a complex dynamic task such as running, we chose to use a relatively simple quantification of the architectural gear ratio. As originally proposed by Brainerd & Azizi (2005), the strain of muscle and fascicle were used to calculate the ratio, whereas we defined muscle strain as the projection of the fascicle length in the direction of the muscle line of action. Although the development of the architectural gear ratio over time is admittedly not considered with this approach, the index provides information about the relationship of muscle to fascicle length changes during the stance phase. Because fascicles shorten during the whole stance phase, we believe that this ratio appropriately quantifies muscle shortening in relation to fascicle shortening. However, future studies may improve the accuracy of AGR measurements by using higher (>80 Hz) ultrasound sampling frequencies and velocity-based AGR calculations. Furthermore, part of the results relating to contractile behaviour are certainly attributable to changes in muscle activation, which was not assessed in the present protocol and should be examined in future studies.

Despite the limitations of tendon length measurements mentioned above, we choose this method over calculating SEE strain. Although the latter theoretically reflects all SEE, we agree with previous authors arguing that the complexity of the structures and their behaviour reflected with this parameter render its interpretation difficult and certainly no less speculative (Farris et al., 2013; Raiteri, Cresswell & Lichtwark, 2016). Importantly, estimates of tendon length per se likely provide better estimates of tendon adaptations, which was the main focus of this study.

Consistent with a previous study using a plantar flexor training intervention (Albracht & Arampatzis, 2013), running kinematics did not change after the training. Yet training was only systematically conducted on one leg in this study, which might have affected running kinematics in ways that could not be seen with the present methods. Finally, despite an unsystematic selection of subjects, baseline differences were observed between groups. In particular, the higher preferred speed of the training group arguably resulted in different running requirements, e.g., for peak tendon force. The purpose of choosing a self-selected speed was to study the effects of the intervention when running is not constrained, to avoid between-group differences in muscle–tendon interaction. In that respect, the fact that Achilles tendon and fascicle behaviour were not different between groups at baseline, despite differences in speed and in some related kinematic parameters, seems to support the rationale for choosing this protocol. The downside of the group mismatch is that it certainly decreased statistical power on a number of variables.

## Conclusion

This study showed that a training intervention aimed at increasing its stiffness altered its behaviour by reducing recoil. Changes in kinematics of tendon recoil differed after training. Yet these changes did not affect fascicle length change patterns in either the bi-articular gastrocnemius or the mono-articular soleus, despite expected differences in the sensitivity of these muscles to changes in tendon mechanical properties. We suggest that training adaptations alter muscle–tendon interaction in more complex ways than suggested by in-series models, via active regulation of tendon-aponeuroses stiffness and, possibly, via different adjustments in the behaviour of synergistic muscles.

##  Supplemental Information

10.7717/peerj.6764/supp-1 Supplemental Information 1 Matlab file with raw data Data are provided in structures for the stance phase for all variables, subjects and included steps.

## Additional Information and Declarations

### Data Availability

The following information was supplied regarding data availability: The raw data are available in a Supplemental File.
